# Three dimensional rotational angiography for assessment of coronary arteries during melody valve implantation: introducing a technique that may improve outcomes

**DOI:** 10.1007/s12471-016-0931-6

**Published:** 2016-12-08

**Authors:** C. R. Pockett, J. W. Moore, H. G. El-Said

**Affiliations:** Rady Children’s Hospital, San Diego, University of California, San Diego, USA

**Keywords:** Coronary angiography, 3D angiography, Percutaneous pulmonary valve, Coronary compression, Aortic root distortion

## Abstract

**Background:**

Adverse events from Melody valve implantation may be catastrophic. To date a role for three dimensional rotational angiography of the aortic root (3DRAA) during Melody valve implantation has not been established.

**Objectives:**

To describe the role of 3DRAA in the assessment of Melody valve candidacy and to demonstrate that it may improve outcomes.

**Methods:**

All patients who underwent cardiac catheterisation for Melody valve implantation and 3DRAA between August 2013 and February 2015 were reviewed.

**Results:**

31 patients had 3DRAA with balloon sizing. Ten were deemed not Melody candidates (5 coronary compression, 2 aortic root distortion with cusp flattening, 2 RVOT was too large, and 1 had complex branch stenosis and a short landing zone). Of the 21 patients who were Melody candidates, 12 had conduits, 6 prosthetic valves and 3 native RVOTs. In patients with conduits, the technique of stenting the conduit prior to dilation was used after measuring the distance between the conduit and the coronary arteries on 3DRAA. In the Melody patients, we had 100% procedural success and no serious adverse events (coronary compression, tears, stent fracture or endocarditis).

**Conclusion:**

As a tool for case selection, 3DRAA may facilitate higher procedural success and decreased risk of serious adverse events. Furthermore, 3D rotational angiography allows stenting of the conduit prior to dilation, which may prevent tears and possibly endocarditis.

## Introduction

Since percutaneous pulmonary valve implantation was first performed by Bonhoeffer in 2000 [1], its use has continued to expand. However, patients may be excluded because of the risk of coronary artery compression. Conventional two-dimensional angiography, including selective coronary artery angiograms, may not definitively demonstrate coronary artery compression during simultaneous right ventricular outflow tract (RVOT) balloon dilation because the lesion is usually proximal and may be masked by the catheter intubating the vessel [2–4]. Moreover, unsupported dilation of stenotic and calcified conduits may result in ruptures or tears requiring covered stents or urgent surgery [5]. The use of three-dimensional rotational angiography (3DRA) in the implantation of Melody valves has been primarily for evaluation of the RVOT [6]. A role in imaging of the aortic root including coronary arteries in this setting has not been reported. The purpose of our study is to describe the use of 3DRA of the aortic root (3DRAA) and coronary arteries in the assessment of Melody valve candidacy.

## Methods

Sixty-nine patients have had percutaneous Melody valve implantation at our institution since we started performing this procedure in October 2010 until February 2015 (68 in the pulmonary position and 1 in the tricuspid position). Since starting routine 3D rotational angiography in August 2013, 38 patients had a cardiac catheterisation to assess for Melody valve candidacy. Of those 7 were deemed not to be candidates for Melody valves on the basis of a large RVOT prior to balloon sizing and thus did not have a 3DRAA. Even though our focus is on the 31 who underwent 3DRAA, we include the 7 who did not undergo 3DRAA for comparison of radiation dose using the same catheterisation equipment (Table 1). All patients had general anaesthesia and underwent an initial diagnostic catheterisation including a pulmonary artery angiogram.

### Technique of 3DRAA

If the RVOT was deemed adequate from the initial screening pulmonary angiogram, 3DRAA was performed with simultaneous balloon sizing using a size 24 or 29 Amplatzer balloon (AGA Medical Corp, Plymouth, MN, USA) or a low pressure Max LD balloon (Cordis, Endovascular, Bridgewater, NJ) in the RVOT over a Lunderquest (COOK Medical, Bloomington, IN, USA) or a Amplatz Super Stiff wire (Boston Scientific, Marlborough, MA, USA). Right ventricular pacing was performed at 180–200/min with breath hold and an infusion rate of 16–18 cc/second for 6 s of a 60% contrast to 40% saline concentration. We performed pacing even though the cardiac output is stopped during balloon inflation as we found it provides better balloon stability and better filling of the coronary arteries. The images were acquired with a C-arm mounted flat panel biplane angiographic system (Toshiba America Medical Systems Inc.) with non-gated, 190-degree rotational image acquisition. Rendering and post-processing of the rotational images was performed on a Vitrea Workstation (Toshiba America Medical Systems Inc.). After post-processing, the image was rotated until we found the view that showed the shortest distance between the balloon and the coronary artery in question. In some cases, we would start with the 3DRAA with a sizing balloon; if the distance measured was small or we were still concerned we would use the 3D image to select the best angle to display the coronary/balloon relationship, then insert a bigger balloon and do a selective coronary angiogram. In most cases we would abort the procedure based on the 3D rotation alone.

### Technique of stenting the conduit prior to dilation

**Fig. 1:**
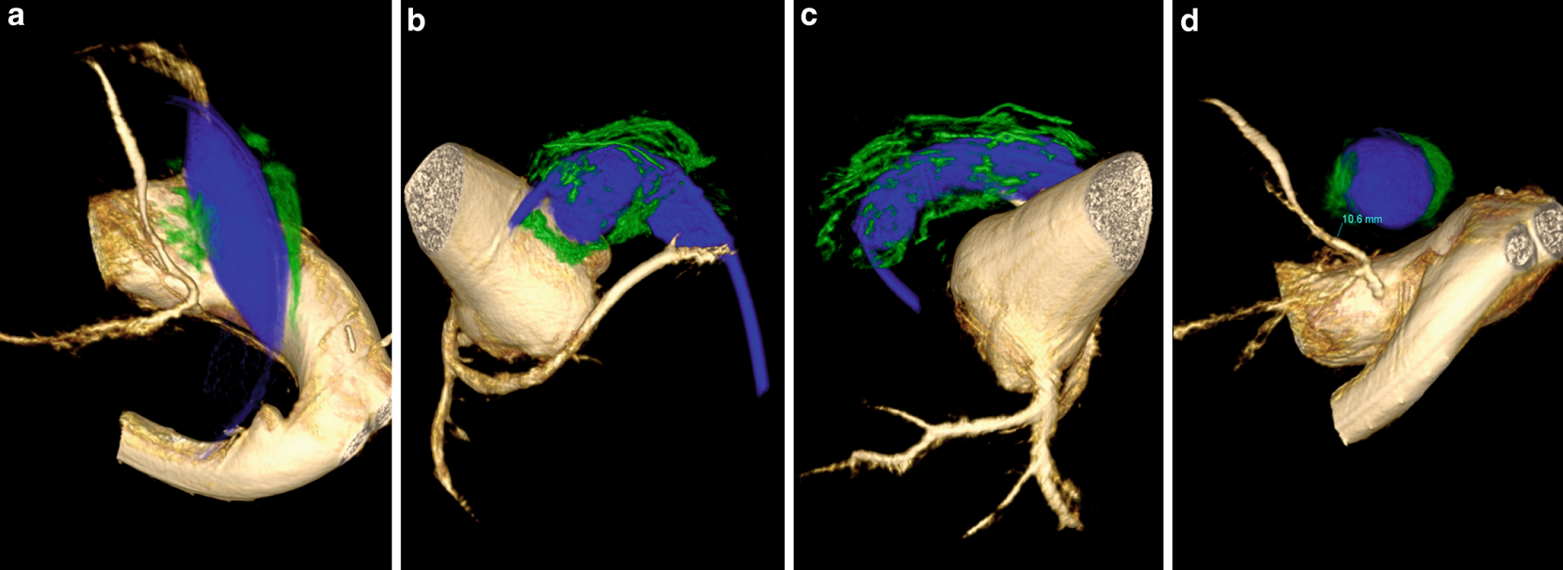
3D rotational angiography demonstrating calcium (*green*) surrounding the balloon (*blue*) in a patient with a single coronary artery. Note the proximity of the calcium to the left anterior descending coronary in panel (**a**, **b**). Calcium surrounds the balloon in panel (**c**). Distance between the balloon and the LAD is measured in panel (**d**)

**Fig. 2:**
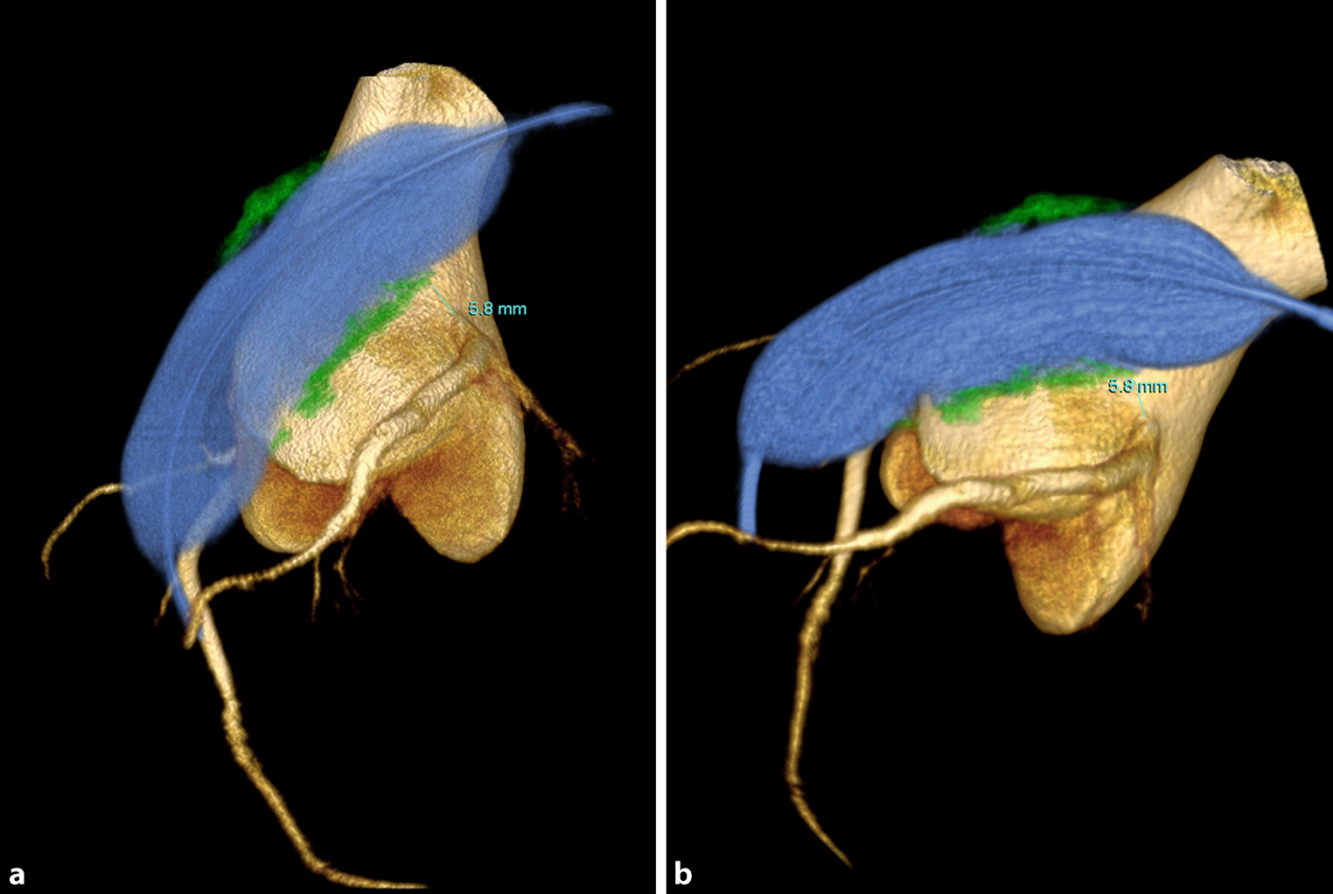
3D rotational angiography (blue: balloon in the right ventricular outflow tract, yellow: aortic root and coronary arteries, green: conduit calcification), demonstrating the distance between the calcium surrounding the balloon and the LMCA in two angled views showing the same measurement of 5.8 mm

In patients with conduits, the technique of stenting the conduit prior to dilation was used. 3DRAA with a sizing balloon was performed, after post-processing, the image was rotated until we found the view that showed the shortest distance between the balloon and the coronary artery in question. The distance between the conduit and the coronary artery in question was measured. Calcium between the conduit and the coronary was assessed on the 3DRAA images (Fig. 1 and 2). A safe distance was calculated based on several factors. We assumed worst case scenario (that the total expansion would occur in this area) and thus used a balloon/stent size that would allow at least 4 mm clearance from the coronary. The amount of calcium was also taken into consideration. For example, if the narrowest area of the conduit was 12 mm and the distance to the coronary in question was 8 mm with no or little calcium, we stented the conduit with a 16 mm stent and if the distance was 6 mm or it was 8 mm with heavy calcium we used a 14 mm stent (Fig. 1 and 2; Table 3). We subsequently re-evaluated the coronary in question and if a distance of more than 4 mm was still present we further serially dilated the stent to achieve the safest maximum diameter for the Melody valve, rather than a predetermined diameter. In some cases, we chose to only implant an 18 mm Melody valve instead of a 20 or 22 if the coronary artery was too close. Palmaz stents (Cordis, Endovascular, Bridgewater, NJ, USA) were used in this group because of their higher radial strength. Several stents are placed in the landing zone until no recoil was observed on fluoroscopy. As adding stents increases the outer diameter, which will get even bigger after implantation of the valve, if there was concern about the distance between the coronary and the balloon, the first stent was placed at a diameter smaller than the desired final diameter and a selective angiogram of the coronary in question was done before enlarging it further. This was done on a case by case basis and depended on how close the coronary artery was.

### Technique for melody valve implant in bio-prosthetic valves

3DRAA and coronary distance were assessed and the usual precautions were taken. Pre-stenting with Palmaz stents was performed; usually only one stent was needed because there is support from the valve stent and fracture is unlikely.

### Technique for melody valve implant in native RVOT

3DRAA and coronary distance was assessed. Pre-stenting with EV3 stents (Covidien/Metronic, Minneapolis, MN) was performed. These stents were selected because their open cell design allows them to conform better to the anatomy than Palmaz stents. Furthermore, these patients generally did not have stenosis, so less support was needed.

### Data collection

Pre-procedure data included age, weight at implant, original diagnosis, past operations, type/size of last conduit or valve placed, degree of conduit stenosis and/or regurgitation by echo and MRI if available. Data collected from the 3DRAA included coronary artery pattern, proximity of the coronaries to the sizing balloon, distance from the inflated balloon to the closest coronary artery, aortic cusp compression, as well as radiation dose for 3DRAA. The study was conducted with approval by the University of California San Diego Institutional Review Board, and patient data collection and storage complied with the Health Insurance Portability and Accountability Act of 1996.

### Comparing radiation dose for 3DRAA with 2D angiography

The radiation exposure during the entire procedure of the 31 patients who underwent 3DRAA was compared with that of the 7 patients who were not deemed to be Melody candidates and only underwent 2D conventional angiograms.

### Statistics

Statistical analysis includes rates of incidence as a proportion. Statistical significance was assessed by Fisher’s exact test and Student’s t‑test for categorical and continuous variables respectively. All tests were two-sided and a *p*-value of less than 0.05 considered significant.

## Results

**Fig. 3:**
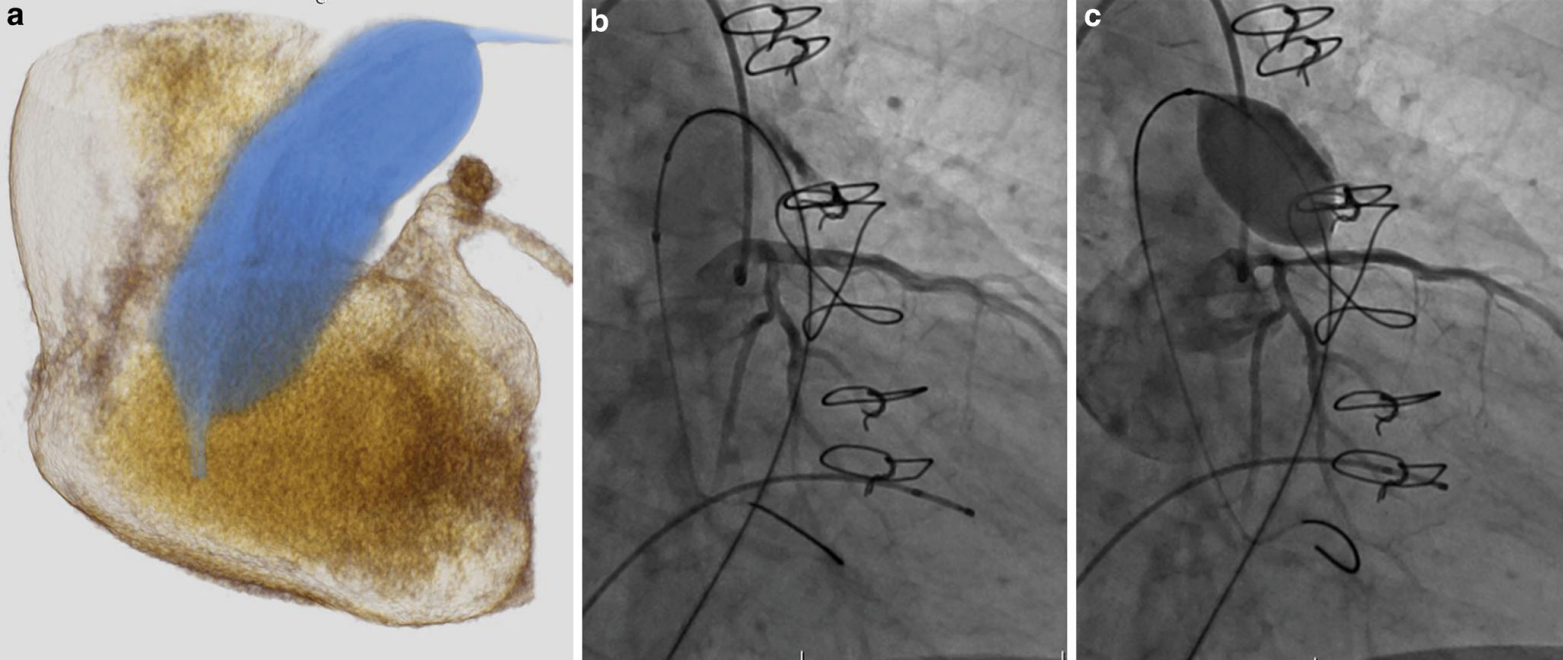
**a** 3D rotational angiogram demonstrating the close relationship of the sizing balloon and the left main coronary artery in a patient with Ross and a dilated aortic root. This was used to determine the best angle to perform the selective coronary angiogram. A selective left coronary angiogram in the angle determined by the 3D rotation with a high pressure balloon 2 mm larger than the waist on the sizing balloon. Note coronary compression with balloon inflated (**c**) compared with with the balloon deflated (**b**)

**Fig. 4:**
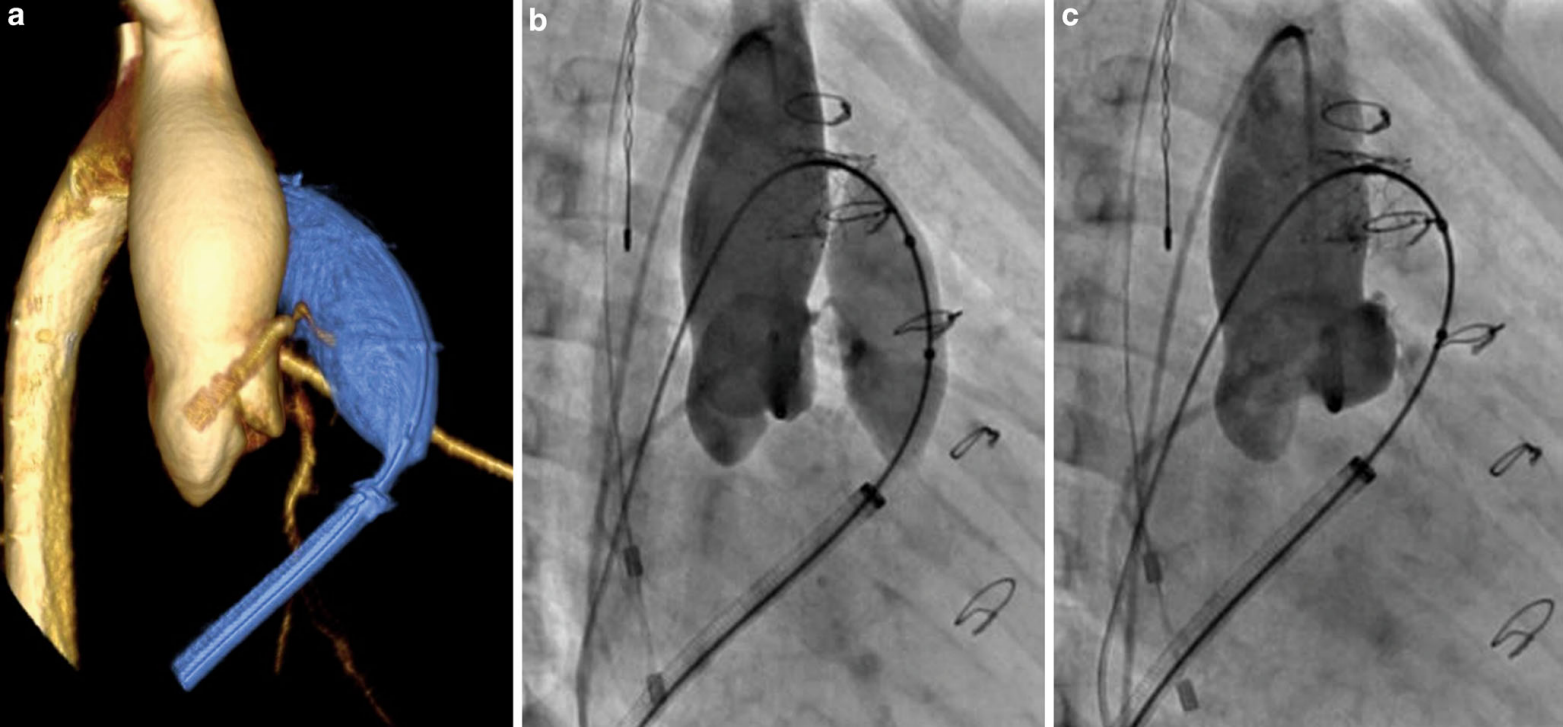
**a** 3D rotational angiogram demonstrating aortic root distortion and coronary cusp flattening, this was used to determine the angle that best showed the cusp compression. An aortogram of the same patient in the angle determined by the 3D rotation demonstrating cusp distortion/compression with the balloon inflated (**b**) and no cusp compression with the balloon deflated (**c**). The right coronary artery appears to be slightly distorted but not significantly compressed with balloon inflated. Patient was turned down for Melody implantation based on cusp compression/distortion

Of the 31 patients who had 3DRAA, 10 were deemed not to be candidates for a Melody valve: 5 because of coronary compression (Fig. 3), 2 because of aortic root distortion with cusp flattening (without coronary compression) (Fig. 4), 2 because the RVOT was too large, and 1 had complex pulmonary artery branch stenosis and a very short landing zone (Table 2). Of the 21 who were deemed candidates for Melody valve implantation, 12 had conduits (Homograft 9, Contegra 3), 6 had bio-prosthetic valves and 3 had a native RVOT with a transannular patch (Table 1 and 3).

**Table 1:** Patient demographics for patients who underwent 3DRA divided in to Melody eligible and ineligible groups

		Ineligible group *n* = 10	Melody group *n* = 21	*P*-value
Sex				0.86
	Male	7	14	–
	Female	3	7	–
Age (years)				1
	Average	15.9	16.6	–
	Median	10	14	–
	Range (min–max)	(3–58)	(5–39)	–
Weight				0.06
	Average	38.5	56	–
	Median	30	50	–
	Range (min–max)	(12.5–86)	(19–125.3)	–
Diagnosis				0.58
	TOF (or Variant)	5	12	–
	Truncus Arteriosus	1	3	–
	Other^a^	4	5	–
Arch side				0.003
	Left	10	12	–
	Right	1	9	–
Type of RVOT				0.0038
	Native	5	3	–
	Contegra	3	3	–
	Homograft	2	9	–
	Bioprosthetic^b^	0	6	–
Indication for PVR				0.269
	Stenosis	3	6	–
	Regurgitation	6	6	–
	Both	1	9	–

*NS* not significant where *p* = <0.05

^a^Other includes: bicuspid aortic valve s/p Ross procedure, severe pulmonary stenosis, pulmonary atresia with intact ventricular septum, atrioventricular canal with pulmonary stenosis, Heterotaxy syndrome with double outlet right ventricle and pulmonary atresia

^b^Bioprosthetic valves include: the Mitroflow Valve, Edwards Perimount Valve, Mosaic Valve, *PVR* pulmonary valve replacement

**Table 2:** Cases in which Melody was not implanted (Melody ineligible)

#	Age (y)	Weight (kg)	Diagnosis	Procedure	#Surg	Conduit type	Size	DysfX	Reason not implanted
1	15	38	TOF/PA	Unifoc/Repair	2	Contegra	18	S	RVOT too large
2	13	31	TA/IAA	Repair/conduit X2	2	Homograft	15	R	RCA compression
3	58	76	Bicuspid aortic valve	s/p Ross	1	Homograft^a^	Unknown	S	LMCA compression
4	8	24.8	d-TGA, VSD	s/p Rastelli	1	Contegra	14	R	Conal branch compression
5	9	52	TOF	s/p repair- no TAP	3	Contegra	22	S	Technical challenge with branch PAs (short MPA)
6	11	30.5	PV stenosis	Multiple PV balloon valvuloplasties	0	Native	NA	R	RVOT too large
7	5	15.7	TOF, PA	s/p TAP	2	Transannular patch	NA	R	RCA compression
8	32	86	TOF	s/p TAP	2	Transannular patch	NA	R	Right coronary cusp flattening and distortion of RCA
9	5	18.2	PA, IVS, ASD	Repair with BT then TAP and Glenn	2	None-Transannular patch	NA	R	RCA compression
10	3	12.5	TOF, LSVC to CS	Repair with TAP	2	None- Transannular patch	NA	S&R	Right coronary cusp flattening and distortion of RCA

^a^Presumed homograft; *ASD* atrial septal defect, *BT* Blalock Taussig shunt, *CS* coronary sinus, *DysfX* conduit dysfunction, *IAA* interrupted aortic arch, *L* left,* LMCA* left main coronary artery, *LPA* left pulmonary artery*, LSVC* left superior vena cava, *MPA* main pulmonary artery, *NA* not applicable, *PA* pulmonary atresia, *PAs* pulmonary arteries*, PV* pulmonary valve, *R* regurgitation, *RCA* right coronary artery, *RVOT* right ventricular outflow tract, *S* stenosis, *s/p* status post, *TA* transannular, *TAP* transannular patch, *TGA* transposition of great arteries, *TOF* tetralogy of Fallot, *VSD* ventricular septal defect

**Table 3:** Technical details of cases that had a Melody valve placed in a conduit to illustrating details of technique of stenting prior to dilation

Age (y)	Dx C, DF	NBD 3D mm	Prox to CA	Dist from CA (mm)	Type/# St	Size BIB	Ball post st	MSz	M	Cath Gr pre	Cath Gr post
29	TA 22,S	10	NA	CR	PZ/3	18	18, 20 Atlas	20	Y-20 Atlas	18	14
36	PS 21,S	17	LM	8.6	PZ/2	22	22 Atlas	22	N	35	12
14	TA 20,S	14	LM	5.8	PZ/3	20	None	20	Y-22 Atlas	45	4
16	TOF 21,S	14	LM	13.8	PZ/3	20	22 Atlas	22	None	32	17
8	TOF 18,SR	14	LAD	10.6	PZ/3	18	18 Atlas	18	None	23	13
13	TOF 21,R	17	RCA	12	PZ/2	22	None	22	None	7	11
13	TOF UK,SR	12	RCA	9.5	PZ/2	16	16,18 Atlas	18	None	15	15
15	TA 20,R	15	RCA	7.4	PZ/2	20	20 Atlas	20	None	17	15
24	TOF 26,R	20	RCA	9	PZ/2	22	None	22	None	7	0
13	TOF 16^a^,S	12	NA	CR	PZ/2	20	20 Atlas	20	None	36	14
23	TOF 22^a^,SR	15	NA	CR	PZ/1	22	None	22	None	35	13
11	DORV 18^a^,SR	12	LAD (SC)	8	PZ/3	16	None	18	Y-18 Atlas	29	16

^a^Contegra, all others were homografts;* Ball post st* balloon used post stent placement,* BIB* balloon in balloon*, BPM* balloon post Melody implantation, *C* conduit size, *DF* conduit dysfunction, *Dist from CA* distance from coronary artery, *DORV* double outlet right ventricle, *Dx* diagnosis, *Gr* gradient, *LAD* left anterior descending, *LM* left main, *MSz* Melody size, *NA* not applicable, *NBD* narrowest balloon diameter, *Prox to CA* proximal to coronary artery, *PS* pulmonary stenosis, *PZ* Palmaz stent, *R* regurgitation, *RCA* right coronary artery, *S* stenosis, *SR* stenosis and regurgitation, *St* stent, *TA* truncus arteriosus*, TOF* teratology of Fallot, *UK* unknown

Of the 21 patients deemed to be eligible for a Melody valve, all underwent successful Melody valve implantation (100% procedural success). There were also no serious adverse events: i. e. no patients had coronary artery compression, conduit tears or conduit ruptures. In selected patients in which the distance to the coronary artery was in question, we elected to use an 18 mm valve instead of a 20 or 22 mm. Despite this conservative approach, the 3 patients who received an 18 mm valve had no significant residual gradients (13,15 & 16 mm Hg).

At follow-up, all of the implanted Melody valves remain in situ with no patients to date requiring explant or re-intervention on their valve. There have been no cases of infectious endocarditis, no cases of stent fracture in this cohort, and no significant re-stenosis or regurgitation after an average of 1.2 years follow-up (range 0.9 to 1.8 years).

When comparing the group that received 3DRAA (*n* = 31) and the one who only received 2D conventional angiography (*n* = 7), we observed that the relationships of radiation exposures between the two imaging modalities differed based on patient weight. Sub-analysis taking patient weight into account showed that for patients weighing less than 50 kg, the radiation exposure for 3D rotational angiography (expressed in dose area product cmGy*m^2^) was higher than that for the 2D angiography (*p* = 0.00782). Conversely, for patients heavier than 50 kg, there was a higher radiation dose area product during 2D conventional angiograms compared with 3D rotational angiograms (*p* = 0.03821).

## Discussion

In this report, procedure success was 100% among patients selected for Melody valve implant using 3DRAA. Furthermore, in these patients, there were no serious adverse events during the procedures or in the short to medium term follow-up. Although our case series is small, we believe that the methodology described in our report provides a more rigorous selection process and allows procedures to be performed more safely than do the conventional methods. [3, 13].

Our candidate selection rate for Melody valve implantation was 73%, which is somewhat lower than in previous reports (83%) [2]. We believe that the lower selection rate was the result of several factors: first, we evaluated a wider range of patients than those specified in the US Food and Drug Administration (FDA) labelling. Second, we relied on 3DRAA for the assessment of potential coronary compression rather than on conventional angiography. Third, in addition to the typical eligibility criteria, related to vessel size and coronary artery compression, we also eliminated patients because of likely significant aortic root or cusp distortion.

Our early patients met the selection criteria defined by the device’s FDA cleared labelling. These patients had failed or failing right ventricle to pulmonary artery conduits and were generally nearly adult sized. However, as our experience grew the range of patients we considered expanded. We evaluated and are reporting here patients with a native RVOT (*n* = 12), only three of which had an RVOT that was suitable for Melody valve implant. In addition, we evaluated very small patients for Melody valve implantation, our smallest patient weighing only 12.5 kg.

The rate of disqualification for Melody valve implantation because of coronary artery compression in our series since we began relying on 3DRAA assessment of coronary arteries was 13% (5/38). This is higher than the previously reported rates of 1 to 6%. [2, 3, 7–9]. Abnormal coronary artery patterns present higher risks for coronary artery compression [10]. However, none of our disqualified patients had such coronary artery patterns. We believe that our method of assessing the risk of coronary artery compression by 3DRAA is more conservative and may provide a greater safety margin than the conventional methods. Our belief is supported by recent studies in patients with coronary artery disease which suggest that the use of 3DRAA may provide assessment of coronary artery lesions, which is superior to assessment by conventional coronary angiography [11–15].

As more sophisticated coronary artery imaging techniques emerge, there may be risk that patients will be disqualified for Melody valve implantation based on anatomic findings of questionable clinical significance. We believe that the existing reports demonstrate the need for improved coronary assessment. Several series, including the sentinel US FDA clinical trial [17], contain anecdotal patients which demonstrate clinical coronary artery compression after valve implant. There are also notable cases of patients whose coronary arteries were evaluated using conventional methods, who presented with clinically significant coronary artery compression up to one-year post implantation [10]. Furthermore, implant procedures are performed in the artificial state of general anaesthesia with controlled heart rates and low cardiac outputs. Certainly in patients who are awake and exercising the aorta and the pulmonary artery increase in size and coronary circulation may undergo compromise not experienced under anaesthesia. For these reasons, we believe that the more conservative and exacting standard offered by use of 3DRAA provides a safer and a more appropriate assessment of susceptibility to coronary compromise than does conventional coronary angiography.

Clearly, even using 3DRAA, there are borderline cases. In such cases, we have usually elected to implant the valve at the smallest nominal diameter (18 mm) and have avoided upsizing to 20 or 22 mm in spite of small residual post-implant gradients (13–16 mm Hg). We believe that this practice may provide an additional margin of safety, and in such patients, mild residual stenosis is well tolerated and allows for clinical improvement over pre-implant clinical status.

A further criterion we have used to assess eligibility for Melody valve implantation is aortic root and cusp flattening or distortion. This is well assessed by 3DRAA, and not assessed by conventional coronary or aortic angiography. We disqualified two patients because we were concerned that distortion or cusp flattening might cause long-term sub-critical coronary insufficiency and lead to ventricular dysfunction. We believe that patients reported with dynamic coronary artery compression during exercise following Melody valve implantation support this concern [10]. In addition, subtle distortion of the aortic root may also lead to aortic valve insufficiency and dysfunction [16]. For these reasons, our current practice is to disqualify patients with significant aortic root compression for Melody valve implantation.

Turning to technical aspects of Melody valve implantation, in our experience, standard use of 3DRAA exposes patients to similar doses of angiographic contrast and radiation as do standard aortic and coronary angiography. For 3DRAA, we use a ratio of 60% angiographic contrast to 40% saline. This allows higher volume injections with less contrast. We have found the images are clearer and easier to clean up when using this formula. Also, only one 3D image is required, as opposed to the multiple images which may be needed in standard angiography. Finally, we have found that pacing induced hypotension, as well as balloon RVOT occlusion combine to provide much cleaner images of the aortic root and coronary arteries. One additional factor here is the very large aortic roots characteristic of tetralogy of Fallot and truncus arteriosus, which must be defined by the 3DRAA.

In our series, the total radiation dose was not different between the cases using 3DRAA and the conventional 2D imaging approaches. Other studies also confirm that use of 3DRAA does not increase overall radiation exposure to patients over use of conventional angiography [11–15]. Interestingly our experience suggests that in patients weighing more than 50 kg, there is actually less radiation exposure used to acquire the 3D rotational angiograms as compared with conventional angiography.

Since 2013, 3D rotational angiography provided the principle tool in assessment of Melody valve candidacy in our institution. This imaging modality offers detailed coronary artery assessment in all planes. Once the rotational image is acquired it can also be employed to determine the best working angle for evaluation of the coronary artery in question during balloon dilation or stent implantation. Additionally, more precise assessment of the coronary artery course and distance to the RVOT/conduit allows for safer Melody valve implantation. 3D rotational angiography also provides a 3D assessment of the calcification patterns throughout the RVOT, which we also factor into our assessment of areas at risk for tears or ruptures.

An additional benefit of routine use of 3DRAA is that it allows us to stent the conduits early in the valve implant procedure at a small diameter, prior to serial high-pressure balloon dilation in preparation for valve implantation. This can only be done after accurate 3D understanding of the coronary artery anatomy and precise measurement of the distance from the area of narrowing and the coronary artery in close proximity is performed. This approach of early stenting of the conduit is inverted from the more standard approach of initial high-pressure balloon dilation of the conduit until the desired Melody implant size is achieved and the stent landing zone is laid down [8, 17–19]. The advantage offered by our technique is that conduit walls are stabilised by the stent, tears or disruptions are likely to be more limited, and placement of covered stents to abort a crisis is less complex. We believe that our approach maximises the structural integrity of the conduit and minimises the risk of major conduit tears and ruptures from initial balloon dilation. In our limited experience, we have not had a major or catastrophic conduit disruption to date, whereas this complication has been reported in 1.4–2.7% of patients in other case series [19–21]. Certainly, the absence of this relatively rare complication may be because of our relatively small patient cohort. However, we advocate this method from first principles and urge others to consider adopting it.

## Conclusion

Three dimensional rotational angiography appears to be a valuable tool for case selection among Melody valve candidates. It may facilitate higher procedural success and reduce the risk of serious adverse events. Furthermore, 3D rotational angiography allows stenting of the conduit prior to dilation, which may prevent tears and possibly endocarditis. Additional studies are needed to confirm our findings.
